# Centenarians: a model of immune resilience against multimorbidity

**DOI:** 10.1093/lifemedi/lnaf033

**Published:** 2025-10-13

**Authors:** Xiaolin Ni, Tingwei Chen, Yuexuan Long, Yuyan Li, Zhiyue Zhang, Erping Long

**Affiliations:** State Key Laboratory of Respiratory Health and Multimorbidity, Institute of Basic Medical Sciences, Chinese Academy of Medical Sciences and Peking Union Medical College, Beijing 100005, China; State Key Laboratory of Respiratory Health and Multimorbidity, Institute of Basic Medical Sciences, Chinese Academy of Medical Sciences and Peking Union Medical College, Beijing 100005, China; State Key Laboratory of Respiratory Health and Multimorbidity, Institute of Basic Medical Sciences, Chinese Academy of Medical Sciences and Peking Union Medical College, Beijing 100005, China; State Key Laboratory of Respiratory Health and Multimorbidity, Institute of Basic Medical Sciences, Chinese Academy of Medical Sciences and Peking Union Medical College, Beijing 100005, China; State Key Laboratory of Respiratory Health and Multimorbidity, Institute of Basic Medical Sciences, Chinese Academy of Medical Sciences and Peking Union Medical College, Beijing 100005, China; State Key Laboratory of Respiratory Health and Multimorbidity, Institute of Basic Medical Sciences, Chinese Academy of Medical Sciences and Peking Union Medical College, Beijing 100005, China

Multimorbidity, defined as the simultaneous presence of two or more age-related diseases within an individual, represents an increasing global concern that carries substantial consequences for individuals, caregivers, and society at large. Currently, the investigation and management of multimorbidity are hindered by several significant challenges, including the complexity of disease combinations and their intricate interactions, individual heterogeneity, the high prevalence of adverse drug events resulting from polypharmacy, and suboptimal treatment adherence [1]. As a result, addressing and treating multimorbidity necessitates a long-term strategy.

Recent research indicates that the deterioration of the immune system associated with aging (called “immunosenescence”) may significantly contribute to the decline of other organ systems, thereby facilitating the onset of numerous age-related diseases due to its role in promoting a low-grade, sterile chronic inflammation. Consequently, age-related diseases often manifest as multimorbidity, ultimately culminating in organ failure and increased mortality risk [2]. Nevertheless, given the complex and systemic nature of human physiology, it remains unclear the intricate interactions between immunosenescence and these diseases. Immunosenescence serves as a significant risk factor for the development of diseases, while certain diseases may expedite the progression of immunosenescence, creating a vicious cycle (Fig. 1A). This interaction forms a complex pathological network, making it a significant challenge to research and treat multimorbidity. In addressing this issue, could a shift in our approach potentially lead to a breakthrough by focusing on positive factors? Specifically, by researching a unique population characterized by optimal health and longevity, we can establish a standard reference for a healthy immune microenvironment that is applicable to the broader population. This reference can facilitate timely adjustments to the immune status of elderly individuals suffering from various health conditions.

**Figure 1.**
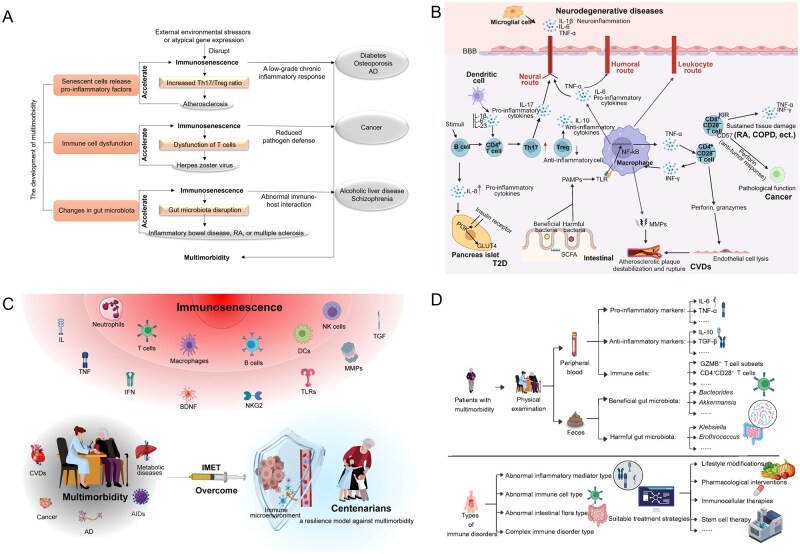
Current status and future treatment strategies. (A) The interaction network between immunosenescence and aging-related diseases forms multimorbidity. Immunosenescence contributes to the development of various diseases, while certain diseases may further exacerbate the process of immunosenescence, thereby creating a detrimental feedback loop. To elucidate this phenomenon, we present three distinct examples: the release of pro-­inflammatory factors by senescent cells, immune cell dysfunction, and changes in gut microbiota. The release of pro-inflammatory factors by senescent cells: Senescent cells are known to secrete pro-inflammatory factors, which can be influenced by external environmental stressors or abnormal gene expression, thereby disrupting the equilibrium of immunosenescence. Immune cells facilitate the release of inflammatory mediators into the extracellular spaces and bloodstream, which can affect the progressive aging of various tissues or organs. This occurs through modifications to the tissue microenvironment or via circulatory interactions, ultimately contributing to the development of multimorbidity. For example, atherosclerosis patients show an increased Th17 to Treg cell ratio, leading to chronic inflammation associated with age-related diseases like diabetes, osteoporosis, and Alzheimer’s disease (AD) [2, 5]. Immune cell dysfunction: Immunosenescence leads to immune cell dysfunction, characterized by a reduction in the diversity of T cells and B cells, as well as a diminished capacity for macrophage phagocytosis. These alterations compromise the body’s ability to eliminate pathogens, thereby heightening the susceptibility to infectious diseases, such as the herpes zoster virus, and to the development of tumors [2, 5]. Changes in gut microbiota: Disruptions in the gut microbiota have been linked to a range of immune-mediated inflammatory diseases, such as inflammatory bowel disease, rheumatoid arthritis (RA), and multiple sclerosis. These alterations can impact the function, proliferation, differentiation, and secretion of immune cells within the intestinal milieu, potentially leading to the onset of additional diseases [2]. (B) The immune characteristics and potential pathogenic mechanisms associated with age-related diseases. Tissue-­resident macrophages get activated by pathogen-associated molecular patterns (PAMPs). PAMPs (infections) engage inflammatory signaling pathways by Toll-like receptors (TLRs), such as nuclear factor-κB (NF-κB). The pro-inflammatory cytokines in turn are promptly secreted and enter the bloodstream, inducing neurodegenerative diseases through three immune-to-brain communication pathways: humoral, neural and leukocyte routes. B cells are critical regulators of inflammation in Type 2 diabetes (T2D) due to their direct ability to promote proinflammatory T-cell function (increased Th17 cells, decreased Tregs cells) and secrete a proinflammatory cytokine profile (an extraordinary inability to secrete the potent anti-inflammatory cytokine IL-10 and an elevated production of proinflammatory IL-8). Pro-inflammatory cytokines enter pancreatic islet cells through islet receptors, affecting the PI3K/Akt signaling pathway and GLUT4 transport, and inducing T2D. Furthermore, Th17 cells are an important subtype of CD4^+^ T cells. Under homeostasis conditions, they mediate the immune response to extracellular bacteria and fungi and maintain the defense function of the intestinal mucosal barrier. However, when the cytokine microenvironment in the body undergoes inflammatory changes, Th17 cells can transform into a highly pro-inflammatory pathogenic phenotype, break through the blood-brain barrier and recruit more immune cells to participate in neuroinflammation, resulting in neurodegeneration. Activated macrophages secrete TNF-α, which is involved in the down-regulation of CD28 by CD4^+^ T cells and the generation of CD4^+^CD28^−^ T cells. Conversely, CD4^+^CD28^−^ T cells secrete a large amount of INF-γ, inducing the activation of macrophages. Macrophages release matrix metalloproteinases (MMPs) that degrade the extracellular matrix, resulting in the destabilization and rupture of atherosclerotic plaques. CD4^+^CD28^−^ T cells mediate direct lysis of endothelial cells and possibly vascular smooth muscle cells by releasing cytolytic enzymes (perforin, granzymes). The destruction of endothelial cells, vascular smooth muscle cells and extracellular matrix can also lead to the destabilization and rupture of atherosclerotic plaques, thereby causing acute coronary events. CD8^+^CD28^−^ T cells are often associated with the lack of perforin, rendering them ineffective Ag-specific killers in chronic viral infections. On the other hand, in certain disease processes such as chronic obstructive pulmonary disease (COPD) and rheumatoid arthritis (RA), they have been reported to express increased levels of cytotoxic mediators, perforin and granzyme B, and pro-inflammatory cytokines, IFN-γ and TNF-α, where CD8^+^CD28^−^ T cells can cause significant damages to normal surrounding tissue in an antigen-nonspecific manner. The structural imbalance of the gut microbiota present in diseases, is characterised by a decrease in the abundance of beneficial genera (such as *Bacteroides*), and an increase in the abundance of harmful genera. Meanwhile, the translocation of bacterial metabolites, such as pathogen-associated molecular patterns (PAMPs), which drive chronic metabolic inflammation via activating the TLR pathway and promoting the induction of proinflammatory cytokines such as THF-α, IL-1β, IL-6, etc. [2, 5, 10]. BBB, blood–brain barrier; CVDs, cardiovascular diseases; KIR, killer cell immunoglobulin-like receptors. (C) Two-layer alterations in immunosenescence and overall scheme of IMET concept between centenarians and multimorbidity. These modifications play a significant role in the occurrence of various age-related diseases and even multimorbidity, ultimately impacting healthy longevity. Centenarians, as a model of immune resilience against age-related diseases, possess unique immune characteristics, which may be a breakthrough to overcome the complex multimorbidity. A novel therapeutic concept—IMET—leverages the distinctive immune characteristics observed in centenarians as a standard for improving the immune microenvironment. AID, autoimmune disease; AD, Alzheimer’s disease; CVDs, cardiovascular diseases; MMPs, matrix metalloproteinases; IL, interleukin; TNF, tumor necrosis factor; IFN, interferon; TGF, transforming growth factor; NKG2, natural-killer group 2; TLRs, Toll-like receptors; BDNF, brain-derived neurotrophic factor; NK, natural killer; DCs, dendritic cells; IMET, immunomicroenvironment therapy. (D) The clinical assessment and therapeutic approach of IMET for individuals with multimorbidity. Upon admission to the hospital, patients with multimorbidity are initially subjected to a comprehensive physical examination. This assessment includes the analysis of peripheral blood and fecal samples to evaluate the levels of inflammatory mediators, the quantity and functionality of immune cells, as well as the composition of the gut microbiota. The findings from these evaluations facilitate the classification of patients into distinct categories of immune disorders. Following this classification, tailored treatment strategies are developed in accordance with the specific type of immune disorder identified in each patient.

## Centenarians as a paradigm of immune homeostasis in the face of multimorbidity

Centenarians are individuals with exceptional longevity and a diverse spectrum of complex phenotypes, characterized by their ability to postpone the aging process and either delay or evade the onset of diseases. This phenomenon is largely attributed to their exceptional adaptability to external environmental factors. Recent findings in centenarians highlight their ability to modulate critical metabolic pathways associated with aging through anti-inflammatory responses, enabling the maintenance of immune homeostasis under environmental stress. This adaptation appears to be a pivotal strategy for achieving maximum lifespan while sustaining health [3]. Therefore, centenarians serve as a model of immune resilience for studying healthy aging and against multimorbidity.

In the cases of inflammatory mediators, evidence suggests that an increased population of effector T cells produces IFN-γ and TNF-α, with TNF-α specifically disrupting insulin signaling pathways. This disruption may lead to insulin resistance, thereby establishing a connection between metabolic disorders and immune responses. Such mechanisms exacerbate the systemic pro-inflammatory state and contribute to the onset of type 2 diabetes (T2D), in addition to impairing cognitive function, ultimately resulting in multimorbidity [4]. However, research on centenarians has revealed that this demographic exhibits increased concentrations of anti-inflammatory molecules, including TGF-β1, IL-10, and IL-1 receptor antagonist, which function to counterbalance the heightened presence of pro-inflammatory molecules such as IL-1β, IL-6, TNF-α, IL-8, CRP, and CXCL9. This dynamic interplay facilitates a state of equilibrium between pro-inflammatory and anti-inflammatory mediators [2].

In terms of immune cell composition, there is frequently a notable elevation in the populations of CD4^+^CD28^−^ and CD8^+^CD28^−^ subsets in the context of age-related diseases. Recent studies suggest that these CD4^+^CD28^−^ T cells, an aggressive and long-lived T cell subpopulation, display characteristics akin to cytotoxic T lymphocytes (CTLs) and natural killer (NK) cells, exhibiting direct cytotoxicity and secreting elevated levels of IFN-γ and cytolytic proteins. CD4^+^CD28^−^ CTLs may contribute to carotid atherosclerotic plaque formation [5]. The expansion of CD8^+^CD28^−^ T cells in cancer patients correlates with cancer staging and poor treatment responses, likely due to the expansion of senescent T cells leading to an inadequate anti-tumor response. Concurrently, the accumulation of inhibitory Treg cells and myeloid-derived suppressor cells may suppress anti-tumor responses through various mechanisms, including the secretion of cytokines such as IL-10 and TGF-β [5]. Recent investigations into anti-tumor therapies have revealed that vaccine-induced CD8^+^ T cells, particularly those expressing granzyme B (GZMB), exhibit significant anti-tumor efficacy in the context of pancreatic cancer treatment [6]. Additionally, a single-cell transcriptomic analysis of Chinese centenarians exhibited an accumulation of GZMB^+^ and CMC1^+^ CD8 T cells (CMC1 is a mitochondrial electron transport chain complex IV chaperon protein) [7]. Similarly, a study utilizing single-cell transcriptomics derived from 7 Japanese supercentenarians and 5 younger controls has revealed a notable increase in cytotoxic CD4^+^ T cells (mean, 25.3% of total T cells in supercentenarians vs. 2.8% of total T cells in controls). Remarkably, CD4^+^GZMB^+^ T cells were quite abundant in the supercentenarians, suggesting a distinct variation in immune cell composition when compared to the general population [7]. It is important to highlight that there has been a notable increase in CD4^+^ CTLs, a finding that is corroborated by research conducted on centenarians in China. Nevertheless, the cellular profile of centenarians in China is predominantly characterized by the presence of CD8^+^ T cells. This discrepancy may be attributed to variations in population characteristics and the methodologies employed in the analyses. Besides, a multimodal integrated analysis of peripheral blood immune cells in centenarians from Europe, Japan, and North America corroborated that the ratio of lymphocytes to myeloid cells, as well as the distribution of non-cytotoxic and cytotoxic cells, underwent changes with aging. Concurrently, significant shifts from CD4^+^ T cell populations to B cell populations were observed in centenarians, indicating a dynamic transition in these immune cells from innate to environmentally induced immune responses [8]. The unique composition of immune cells in centenarians highlights a pattern of immune remodeling characterized by an increased quantity and enhanced functionality of cytotoxic T cells, which may be essential for healthy aging.

In the case of gut microbiota, research has indicated a decrease in both the abundance and diversity of *Bacteroides* in the context of inflammatory bowel disease, obesity, and T2D [9]. Furthermore, the levels of *Bacteroides vulgatus* and *Bacteroides dorei* are also diminished in individuals diagnosed with coronary artery disease compared to healthy individuals. Experimental studies conducted on murine models have demonstrated that *Bacteroides vulgatus* and *Bacteroides dorei* are capable of lowering blood endotoxin levels, decreasing the synthesis of intestinal microbial lipopolysaccharides, effectively suppressing pro-inflammatory immune responses, and ameliorating symptoms associated with atherosclerosis. However, the heightened diversity has been observed in centenarians, along with the increased presence of *Bacteroides fragilis*. It is hypothesized that the enhanced longevity is achieved through the upregulation of the anti-inflammatory cytokine IL-10 [2]. A study of 297 Guangxi centenarians revealed their gut microbiota exhibits high species diversity, reduced potentially inflammatory bacteria, and elevated beneficial bacteria. Predominantly featuring a *Bacteroides*-dominated enterotype, it shows increased species evenness, enriched beneficial *Bacteroidetes*, and depleted potential pathobionts. Unlike centenarians, the general elderly have a higher abundance of potentially harmful/pro-inflammatory symbiotic microorganisms (e.g. *Klebsiella*, *Streptococcus*, *Enterobacter*, *Erysipelatoclostridium*) compared to both centenarians and younger individuals. Longitudinally tracked over 1.5 years, these distinctive microbiome traits in centenarians intensify with age. Collectively, these findings highlight the youth-like gut microbiome aging patterns associated with exceptional longevity [10]. Moreover, cohort studies conducted in areas with a significant population of centenarians, such as Italy and China, have revealed that their intestinal microbiota is characterized by a high abundance of *Ruminococcaceae*, *Akkermansia*, and *Christensenellaceae*, which have been classified as potentially beneficial bacteria. These bacteria are linked to various health parameters, including body mass index, immune regulation, and healthy homeostasis. Notably, a decline in the abundance of *Faecalibacterium*, *Roseburia*, *Coprococcus*, and *Blautia*, alongside an increase in *Enterobacteriaceae*, has been observed in Japanese individuals aged 90 to 100 years. While these microbiota characteristics are similarly to those identified in Italian centenarians, distinctions exist when compared to the microbiota of centenarians in China [2]. These findings suggest that centenarians possess distinctive features in their intestinal microbiota composition. A balanced composition and proportion of gut microbiota are associated with the facilitation of anti-inflammatory processes and health maintenance. Nevertheless, variations may arise across different regions due to environmental factors and dietary practices.

In summary, three factors (inflammatory mediators, immune cell composition, and gut microbiota) may play a crucial role in influencing longevity phenotypes in centenarians through their unique immunosenescence characteristics and may also serve as potential targets of multimorbidity for preventive and therapeutic interventions (Fig. 1B).

## Immunomicroenvironment enhancement therapy (IMET): a new therapeutic concept has been proposed for treating multimorbidity

Currently, the management of immunosenescence has gained significant traction, encompassing a range of approaches including lifestyle modifications, pharmacological interventions, immunocellular therapies, and stem cell therapy [9]. For example, engaging in regular physical activity enhances the functionality of immune cells, decreases the production of pro-inflammatory cytokines such as IL-6 and TNF-α, diminish the prevalence of chronic inflammation, and mitigates the onset of age-related diseases. Research indicates that certain anti-inflammatory medications, particularly non-steroidal anti-inflammatory drugs such as aspirin and ibuprofen, can alleviate systemic chronic inflammation, thereby contributing to the deceleration of the aging process. Metformin has been found to induce autophagy in CD4^+^ T cells, facilitate the transition from an inflammatory to a non-inflammatory state, inhibit the production of pro-inflammatory cytokines, and ameliorate immunosenescence. Additionally, metformin significantly improves the gut microbiota composition in patients with T2D by increasing the abundance of mucinous *Achaemenophilus* and other short-chain fatty acid (SCFA)-producing bacteria. This modulation of the gut microbiota is associated with a reduction in all-cause mortality and a lower incidence of various age-related diseases. Moreover, Chimeric Antigen Receptor T-Cell Immunotherapy (CAR-T), which involves the modification of T cells to express chimeric antigen receptors (CARs) for the specific recognition and elimination of senescent cells, has shown promising therapeutic outcomes in disease models, including liver fibrosis. Mesenchymal stem cells (MSCs) exhibit immunomodulatory and anti-inflammatory properties, capable of inhibiting the excessive activation of immune cells through various mechanisms, thereby alleviating inflammatory responses. The transplantation of MSCs has been shown to improve skeletal muscle function and reduce inflammatory levels in frail individuals [9].

Given the contemporary challenges in research and treatment associated with multimorbidity [1], alongside the intricate interactions between immunosenescence and age-related diseases, it appears that current treatment approaches may be limited to individual diseases or those with well-defined pathogenic targets. Consequently, there is an urgent need to re-evaluate existing paradigms and to develop a comprehensive set of effective therapeutic strategies for multimorbidity. By synthesizing the biological mechanisms underlying multimorbidity, researchers identify the pivotal role of immunosenescence and uncover both the shared and unique immune characteristics associated with age-related diseases and centenarians [8]. This leads us to ask: could the unique immune profiles of centenarians serve as a benchmark for improving the immune microenvironment in patients with multimorbidity (Fig. 1C)?

Therefore, we propose a novel therapeutic concept, immunomicroenvironment therapy (IMET) (Fig. 1D). In contrast to the prevailing immunotherapy strategies that rely on disease classification, the IMET framework directs subsequent therapeutic interventions based on the categorization of the immune performance exhibited by individual patients. Using patients with coronary heart disease complicated by diabetes as a case study, we will detail the specific therapeutic strategy within the IMET framework. Initially, the patient will undergo assessments for inflammatory mediators, as well as evaluations of the composition and function of immune cells in peripheral blood, alongside analyses of intestinal microbiota in fecal samples. Based on the immune performance indicated by these test results, patients with multimorbidity can be categorized into various types of immune disorders, including abnormal inflammatory mediator type, abnormal immune cell type, abnormal intestinal flora type, and complex immune disorder type. If the patient was categorized as exhibiting an abnormal inflammatory mediator profile, lifestyle modifications will be recommended as part of the therapeutic approach. Should the patient be identified as having an abnormal immune cell profile, pharmacological interventions or immunocellular therapies will be implemented. In cases where a complex immune disorder is diagnosed, stem cell therapy will be advised. Overall, the strategic framework of IMET represents a significant departure from conventional immunotherapy approaches.

## Conclusion

The investigation and management of multimorbidity present significant challenges due to the intricate nature of human physiology. The unique immune characteristics observed in centenarians, which facilitate the postponement or avoidance of age-related diseases, provide valuable insights into the interactions between immunosenescence and multimorbidity. This understanding may inform therapeutic approaches such as IMET. However, utilizing centenarians as a benchmark for multimorbidity assessment has limitations: insufficient comparative/quantitative immune analysis relative to multimorbidity prevents comprehensive immune reference indicators; empirical evidence linking immune performance indicators to disease incidence/progression is absent; race/region disparities exist. Future research endeavors should focus on quantifying the immune profiles of centenarians, comparing these profiles with disease-associated characteristics, screening for inflammatory mediators in centenarians, and elucidating the pathogenic mechanisms of their immune responses. Such efforts are essential for effectively addressing multimorbidity and facilitating the translation of IMET. Additionally, China’s proactive strategies for managing an aging population may redirect research efforts towards preventive measures, thereby supporting studies on centenarians. Nonetheless, challenges such as the establishment of specialized outpatient services, insurance reimbursement issues, the need for interdisciplinary expertise, and relevant policy considerations must be adequately addressed.
